# Supplementary material for: A SARS-CoV-2 spike ferritin nanoparticle vaccine protects against heterologous challenge with B.1.1.7 and B.1.351 virus variants in Syrian golden hamsters
Source: bioRxiv. 2021 Jun 16:2021.06.16.448525. Preprint. [Version 1] doi: 10.1101/2021.06.16.448525 (PMC8219092; doi:10.1101/2021.06.16.448525)
Supplement: Supplement 1 [file media-1.pdf]

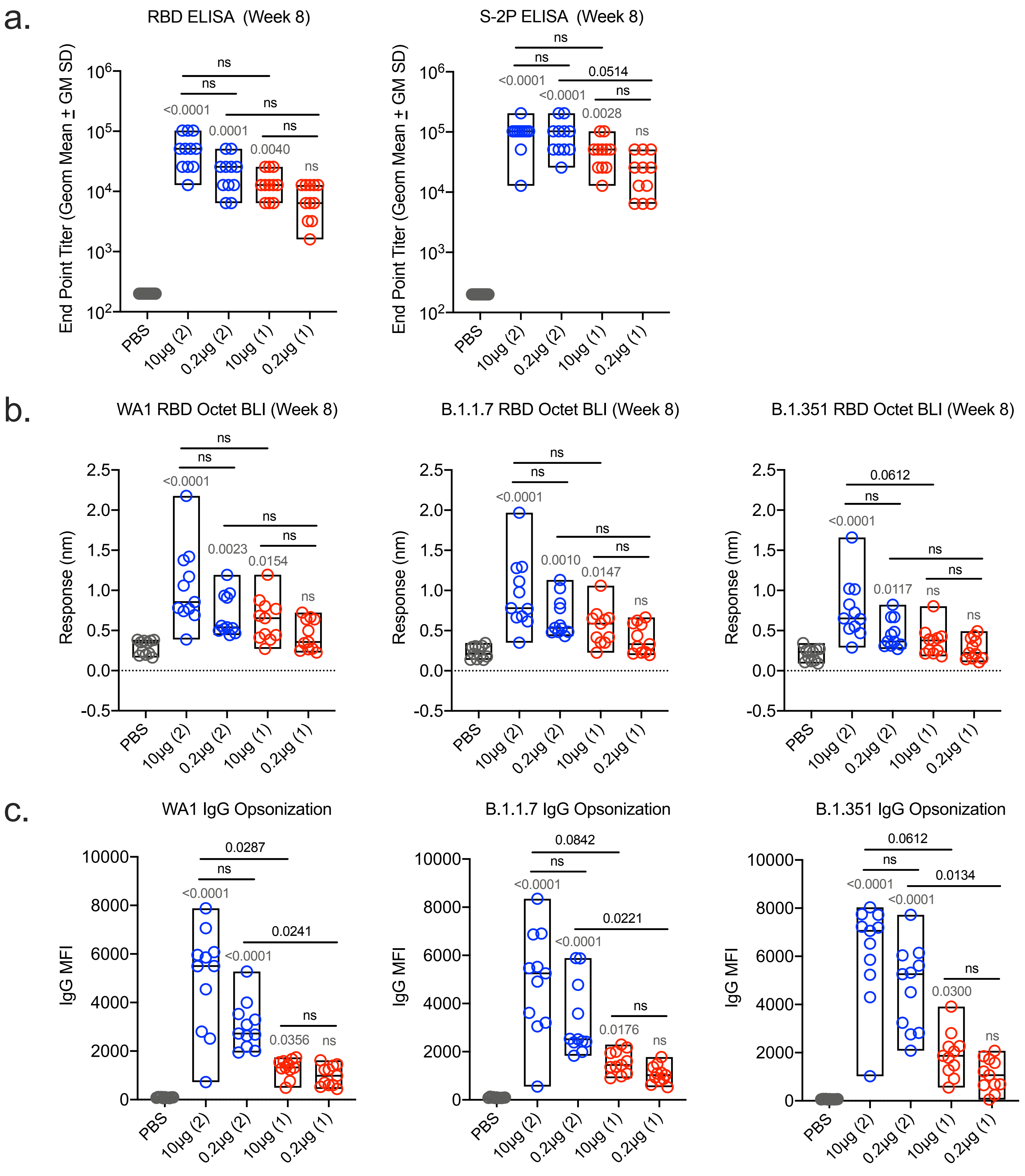
a.
RBD ELISA (Week 8)
End Point Titer (Geom Mean ± GM SD)
10^6
10^5
10^4
10^3
10^2
<0.0001
0.0001
0.0040
ns
PBS
10μg (2)
0.2μg (2)
10μg (1)
0.2μg (1)
S-2P ELISA (Week 8)
<0.0001
<0.0001
0.0028
0.0514
b.
WA1 RBD Octet BLI (Week 8)
Response (nm)
2.5
2.0
1.5
1.0
0.5
0.0
-0.5
<0.0001
0.0023
0.0154
B.1.1.7 RBD Octet BLI (Week 8)
0.0010
0.0147
B.1.351 RBD Octet BLI (Week 8)
0.0612
0.0117
c.
WA1 IgG Opsonization
IgG MFI
10000
8000
6000
4000
2000
0
0.0287
0.0241
0.0356
B.1.1.7 IgG Opsonization
0.0842
0.0221
0.0176
B.1.351 IgG Opsonization
0.0134
0.0300

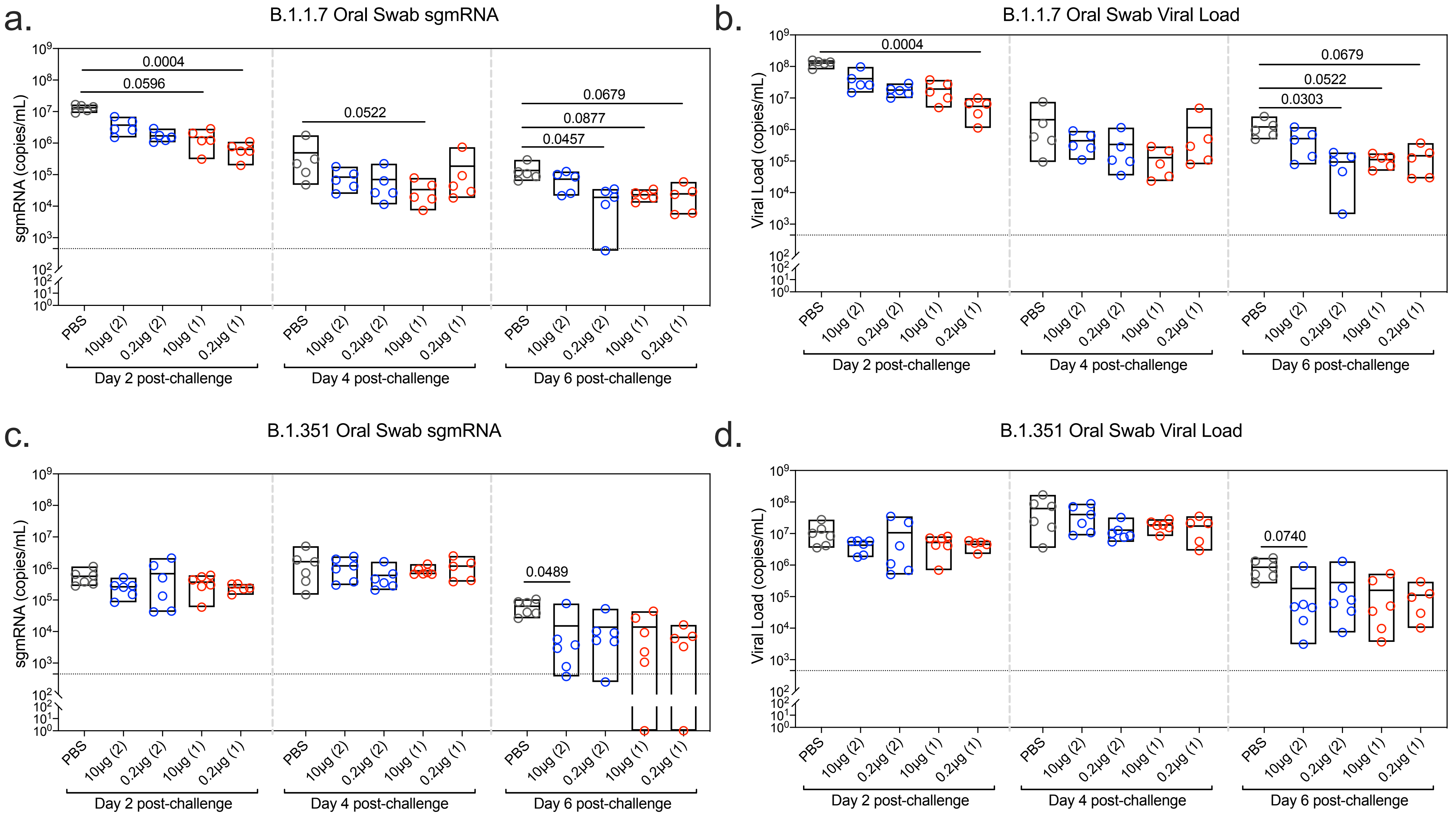

a.
B.1.1.7 Oral Swab sgmRNA
sgmRNA (copies/mL)
0.0004
0.0596
0.0522
0.0679
0.0877
0.0457
PBS
10µg (2)
0.2µg (2)
10µg (1)
0.2µg (1)
Day 2 post-challenge
Day 4 post-challenge
Day 6 post-challenge
b.
B.1.1.7 Oral Swab Viral Load
Viral Load (copies/mL)
0.0004
0.0679
0.0522
0.0303
c.
B.1.351 Oral Swab sgmRNA
sgmRNA (copies/mL)
0.0489
d.
B.1.351 Oral Swab Viral Load
Viral Load (copies/mL)
0.0740

Supplemental Table 1A

			IP Pathology (# observed/total n)				
Challenge Strain	Vaccine regimen	Vaccine dose	None	Minimal	Mild	Moderate	Marked
**B.1.1.7**	Prime-boost	PBS				3/5	2/5
		10 µg (2)	3/5	2/5			
		0.2 µg (2)	2/5	3/5			
	Single	10 µg (1)	2/5	1/5	1/5		1/5
		0.2 µg (1)	1/5	1/5		2/5	1/5
**B.1.351**	Prime-boost	PBS				4/6	2/6
		10 µg (2)	4/6	1/6	1/6		
		0.2 µg (2)	1/6	2/6	1/6	1/6	1/6
	Single	10 µg (1)	1/6	3/6	2/6		
		0.2 µg (1)*		3/5	1/5		1/5

*one animal lost to study due to injury unrelated to infection.

Supplemental Table 1B

			SARS-CoV-2 Viral Antigen per Section (# animals observed/total n)					
Challenge Strain	Vaccine regimen	Vaccine dose	None	Single cell	<5 cells	<20 cells	<40 cells	>500 cells
**B.1.1.7**	Prime-boost	PBS						5/5
		10 µg (2)	3/5	2/5				
		0.2 µg (2)	4/5	1/5				
	Single	10 µg (1)	3/5		1/5	1/5		
		0.2 µg (1)	1/5	1/5	1/5	2/5		
**B.1.351**	Prime-boost	PBS						6/6
		10 µg (2)	5/6			1/6		
		0.2 µg (2)	1/6	5/6				
	Single	10 µg (1)	4/6			2/6		
		0.2 µg (1)*		2/5	2/5		1/5	

*one animal lost to study due to injury unrelated to infection.

Supplemental material

Supplemental Figure 1. Additional antibody responses following SpFN-ALFQ immunization. **a**, ELISA was performed using either WA1 derived Receptor Binding Domain (RBD) or S-2P Spike proteins from sera taken at week 8. The vaccine regimens are indicated on the x-axis by phosphate buffered saline (PBS) control or SpFN dose with the number of vaccinations in the regimen given parenthetically and by color code (blue, 2- dose, red, 1- dose). Endpoint titers are given on the y-axis as geometric mean titers with data displayed in box plots with the top and bottom bars of the box the standard deviation and the middle bar as the median value. P-values for SpFN-ALFQ vaccination groups compared with PBS control are given just above the boxes in light grey while inter- and intra-regimen p-values are given above the boxes in black. ns, not significant ($p > 0.05$). **b**, Octet Biolayer Interferometry (BLI) responses against the WA1, B.1.1.7, and B.1.351 sequences of the RBD are given for the vaccination regimens as in **a** on the x-axis from sera collected at week 8. BLI responses are given in nanometers (nm) on the y-axis. Vaccination regimen color coding and statistical treatments are as in **a**. **c**, IgG opsonization as measured by binding to SARS-CoV-2 Spike protein expressing expi293F cells subsequently stained by fluorescently tagged goat anti-hamster IgG and detected by flow cytometry. Fluorescence is given as mean fluorescence intensity (MFI) on the y-axis. Vaccination regiment color coding and statistical treatments are as in **a**.

Supplemental Fig 2. Quantitative SARS-CoV-2 total and subgenomic mRNA (sgmRNA) viral load from oral swabs following challenge. Oral fluid collection from were obtained by swabbing at days 2, 4, and 6 post challenge in hamsters and submitted to quantitative SARS-CoV-2 total and

sgmRNA analysis. Viral load is given on y-axes at copies/mL of oral fluid plotted on a log10 scale. Vaccination regimen groups, stratified by day post-challenge, are given on the x-axes: Phosphate buffered saline (PBS) control, gray circles; 10 μg and 0.2 μg 2- dose regimen groups, blue circles; 10 μg and 0.2 μg 1- dose regimen groups, red circles. Data are given in box plots where the horizontal bar is the median value for each group. Where values between groups are statistically meaningfully different, p-values are given above horizontal black bars showing the analyzed groups. In all other cases, the p-values were > 0.05. **a**, sgmRNA viral load from B.1.1.7 challenged hamsters; (B) total RNA viral load from B.1.1.7 challenged hamsters; **c**, sgmRNA viral load from B.1.351 challenged hamsters; **d**, total RNA viral load from B.1. 351 challenged hamsters.

Supplemental Table 1. Semi-quantitative histopathology and IHC scores

Lung tissues were collected at necropsy on day 6 post-challenge, fixed with neutral buffered formalin, and stained with hematoxylin and eosin (H&E) for standard microscopic examination as well as submitted for immunohistochemical (IHC) staining for SARS-CoV-2 nucleocapsid (N) protein. (A) H&E sections were semi-quantitatively scored on the histopathology present in all lung sections examined (Methods). The overall severity of interstitial pneumonia (IP) present in individual animals in the PBS control and vaccinated groups is given. (B) IHC sections were examined at 400X magnification, and evaluated for the number of immunopositive cells per slide. Distribution of the number of immunopositive cells per section is given per PBS control and 2- dose and 1- dose vaccine group. The number of vaccinations per vaccine group are given parenthetically.
